# Hydrophobia

**Published:** 1855-05

**Authors:** 


### EDITORIAL DEPARTMENT.

### HYDROPHOBIA.

The people residing in various localities around us, have been recently thrown into a state of painful alarm in consequence of the appearance among them of dogs, which, from their conduct, were supposed to be rabid. Rumors of an exciting character were followed by others still more horrifying; animals had been bitten by them that afterwards became rabid; and persons were also bitten, who were expected to go mad. Dogs were killed by scores, and every cur, whose caudal extremity from an unfortunate longitude happened accidentally to fall between the nether extremities, was looked upon with suspicion, and if a little froth or mucous flowed from his mouth, it proved his death-warrant—signed, sealed, and soon executed.

These are the usual concomitants of the cry of "*mad dog*" in community, and yet, from the horrid nature of the disease, it is not to be wondered that the public mind should be tortured to the extremity of fear and excitement, and commit, under this state of things, rediculous excesses and indiscretions.

We have thought we could not more appropriately occupy our time or space than by offering a few thoughts on the subject. And we do this the more readily, because we are aware there are abroad among the people, and even among some medical men erroneous views in relation to the disease and its prevention.

The poison when introduced into the system is usually through an abraded surface made either by the teeth of the dog or some open surface previously existing, as a wound or ulcer with which the poison comes in contact. And yet it is supposed that the mucous lining of the mouth would absorb poison if brought in contact by a kerchief in wiping the mouth. Of this there are doubts however.

The symptoms are peculiar to itself. There is first a feeling of numbness in the original wound and a tingling sensation proceeding from it along the course of the limb. Sometimes the cicatrix opens up afresh and gives out a sanies discharge, attended with a hot burning sensation. This constitutes the first stage which may last for several days. This is succeeded by mental symptoms characterised by great depression of spirits, irritability, and an unconquerable fear of impending danger. This is the second stage, and is very soon followed by others of a still more alarming character.

Now there is stiffness of the throat and of the neck, manifestly tetanic symptoms; the attempt at swallowing is done in a hurried and excitable manner, and when done is performed with difficulty. A current of cold air, the splashing of water on the sides of a vessel, or the least noise aggravate the symptoms. This is followed by violent spasms which continue seldom beyond seventy-five hours, and death closes a scene of anguish and suffering beyond the pen to describe.

We have said that we have tetanic symptoms as indicated by the stiffness of the throat and neck. It is then of the utmost importance to be able to make a correct diagnosis. Let us for a moment examine the pathology of each. In hydrophobia there is an abnormal sensibility of the spinal marrow. In tetanus the spinal marrow is *equally* excitable; but in this case, the spasms are excited, and kept up by a constant source of irritation as by an injured nerve or one in a state of irritation. In the former this is not the case, and therefore the spasms will depend upon excitation from without, as for instance a stream of cold air, a sudden noise, &c. The one has its source in the blood, the other in an irritated or wounded nerve. In one there is spasam and then remission, in the other the spasm depends for its return upon circumstances. This is the distinction. There is a condition of the spine similar to that condition of erethism produced by strychnine both in tetanus and hydrophobia according to the opinion of Marshall Hall. In the one case the blood supplies the material for the production of this erethism, in the other the source of excited exaltation is a local one producing its general pathological results. In the one case an occasional exciting cause will awaken spasms, and thus the erethism is momentarily diminished to be soon re-supplied by the material in the blood, in the other it is expended to be supplied through the exciting agency of an ever present irritation of the injured or irritable nerve.

The anatomical characters of this disease are not very satisfactory, for, as Wood says the "brain and spinal marrow are both perfectly sound" in some cases. But in a large majority of cases, according to the observation of others, there is a vascular turgesence of its meninges. But there is an altered condition of the spinal centre, and this change consists in an exaltation of *sensibity* or an extreme *susceptibility.* This is induced by the poison, and doubtless, through the agency of the blood just as the brain is poisoned by certain matters, as from urea, carbon, &c. Each spasm is an excited reflex action, and in this respect M. Hall maintains that it resembles the effects of strychnine.

On this account he avers that death in hydrophobia takes place from *asphyxia*, and that if we succeed in curing the patient, we must avoid the spasm in which there is a paroxysmal closure of the larynx. If the asphyxiated condition can be avoided, then he maintains that the patient has no fear of death. But it will be seen from this, that the indication first would be to prevent spasm, and if we can avoid this, then we also avoid the asphyxia. The one follows the other in the order of sequence; but if the spasm continue, what is then to be done?

The spasm, he says, may be avoided by placing the patient on a spring bed, so as to avoid sudden concussions, and the bed to be surrounded so as to prevent the exciting impressions of cold air. Every current of air, every shake of the bed, and all emotional influences, should be controlled in order that the spasm may be avoided. Now what is to be done if these spasms continue? He recommends tracheotomy, and if this be performed and the asphyxia be avoided, then he asks the question, "Why should he not survive until the poison should be eliminated from the system?" If he would not die of nervous exhaustion—and we would have no fear of this—we cannot ourselves see why he should die from this disease. The suggestion is valuable, and should be adopted as all measures heretofore have proved unavailing, except a few recent cases which were relieved by chloroform.

A case is reported in the London Lancet, for 1848, July, in which chloroform proved effective in a case of hydrophobic mania. A case was also treated by it in Camden, New Jersey, in which it proved effectual. There were strong doubts of this case, however, as the lady affected was subject to hysterical spasms, which it sometimes simulates, and besides this, there was no certainty that the dog which inflicted the wound was rabid.

The results in the treatment of hydrophobia heretofore, leave us with no hope that any of the remedies which have been used will prove of any value unless the plan proposed by M. Hall or the chloroform shall prove effective on a further and more extended trial. Opium, mercury, strychnia, prussic acid, hellebore, cevadilla, galvanism, electricity, vapor baths, even the poison of vipers, and indeed the whole category of medicines has been run over, and nothing has yet been discovered which is entitled to the consideration of a remedy or to rank as such. Bleeding *ad deliquium*, and the cold bath have succeeded about as well as anything else.

Preventative measures have been adopted and numerous remedies have been used for that purpose. The manner in which certain remedies, secret and known, have acquired a reputation arises from the fact that nineteen out of every twenty dogs which have been reputed as mad and which too have inflicted bites, have not been rabid. Chick weed and the far famed *mad stone* will do as well in such cases as anything else, and because they have proved successful in nineteen cases out of twenty where no rabid virus had been imparted, they were therefore *deified* as remedies. But in cases of true rabid poison they are not only useless but *worse* than useless for the obvious reason that the faith reposed in them induces the injured to rely upon them to the exclusion of remedies which experience has proved effectual. The public mind should be undecieved on this subject, and those journals which reach the populace, should place the truth before them, and save them from being misled by abandoned charlatans and reckless emperics who for a consideration, and that too often very small, are willing to sacrifice the health of their fellows, or cheat them into an exposure of their lives.

If there be good grounds to suspect that a person has been bitten by a rabid dog, the surest prevention lies in excising, as soon as possible, the whole surface exposed. This should be done effectually; the trace of every tooth followed, and the surface injured by it removed by the knife. If a large vessel lie in the way then cut where you can and apply caustic to the balance or inject with the strong solution which will find its way to the entire mutilated surface. The celebrated English Surgeon, Bransby Cooper, than whom there is no better authority, recommends that a strong solution be injected into deep ragged wounds first, before using the knife, as this instrument will find it a guide as it progresses, and then again after this, the solution of the nitrate can be used. If this be done effectually and immediately after the bite, there is very little danger, he maintains, of hydrophobia succeeding. There are cases in which the doubt of the rabid condition of the animal is very strong, and where the person bitten is excitable, physically and mentally, that the operation would be scarcely justified. Dogs will bite often from retaliation, even their masters to whom they are devoted, in which case there is no virus. The animal, however, in all doubtful cases, should be kept alive, but secured, in order to test whether they are rabid or not. If they prove to be so, then we must prepare for the worst, but if not, the minds of both patient and friends are set at rest.

Hydrophobia usually occurs after successful introduction of the rabid poison in from eighteen to ninety days. The time that elapses between its introduction and the symptoms which usher in the attack, is called the period of incubation. Doubtless, in many cases, it wears out, or finally expends itself, in others again, it requires some exciting cause or causes to develope it. It has appeared, it is stated upon good authority, in twelve years after the subject has been bitten, but this is very doubtful. Dr. Wood accounts for its appearance under such circumstances, upon the probability that the poison may have been absorbed by the mucous membrane of the mouth, brought in contact by a handkerchief or the hand itself, from animals not suspected of being rabid.

The subject is a grave one, and does not wholly belong to the medical man. The number of the canine race is vastly increased beyond the requirements of vigilence or the necessities for protection. State and municipal regulations should be adopted to prevent excesses in numbers, and thereby avoid danger. If one of the species be attacked, it then would spread with great rapidity. In towns and cities, dogs are scarcely required at all for protection, and they are *there* required for no other purpose. If in a crowded community where there is but one to every family, and one of these should become diseased, it would spread with great rapidity, and the consequences might be of the most frightful character. Prudent counsels would suggest the propriety of levying a heavy tribute upon those who will, in view of the dangerous consequences to community, continue to keep upon their premises, an useless, and often dangerous animal.

Excessive heat and cold favor its production, and both of these extremes we have had within the limits of twelve months, and therefore it is not a subject of wonder that numerous cases should appear through the country. In our own city, the *canine* race equals in numbers the population, almost, of the higher race, the genus *homo*, which fact in our humble opinion demands, for more reasons, but of less grave import than those above rererred to, that our "*fathers*" should adopt measures for a speedy reduction of the numbers or what would better comport with the public safety, *a total annihilation* of the race within our limits.

